# Under the Hood: Understanding the Features of Mucin in Pseudomyxoma Peritonei

**DOI:** 10.3390/jcm12124007

**Published:** 2023-06-12

**Authors:** Pedro Villarejo-Campos, Mariano García-Arranz, Siyuan Qian, Santos Jiménez de los Galanes, Víctor Domínguez-Prieto, Juan Felipe Vélez-Pinto, Ismael Guijo Castellano, Montiel Jiménez-Fuertes, Héctor Guadalajara, Damián García-Olmo

**Affiliations:** 1 Department of Surgery, Fundación Jiménez Díaz University Hospital, Avda. Reyes Católicos, 2, 28040 Madrid, Spain; siyuan.qianv@quironsalud.es (S.Q.); santos.jimenez@quironsalud.es (S.J.d.l.G.); victor.dominguez@quironsalud.es (V.D.-P.); felipevelezpinto@gmail.com (J.F.V.-P.); iguijo@fjd.es (I.G.C.); montiel.jimenez@quironsalud.es (M.J.-F.); hector.guadalajara@uam.es (H.G.); damian.garcia@uam.es (D.G.-O.); 2 Department of Surgery, Universidad Autónoma de Madrid, C/Arzobispo Morcillo s/n, 28034 Madrid, Spain; mariano.garcia@quironsalud.es; 3 New Therapies Laboratory, Health Research Institute-Fundación Jiménez Díaz University Hospital (IIS-FJD), Avda. Reyes Católicos, 2, 28040 Madrid, Spain

**Keywords:** pseudomyxoma peritonei, mucin, MUC-2, microbiome, pseudomonas

## Abstract

Pseudomyxoma peritonei (PMP) is a rare malignant growth characterized by the production of mucin and the potential for peritoneal relapse. This study aimed to investigate the immunohistochemical and biological characteristics of mucin in patients with cellular and acellular PMP. We prospectively analyzed mucin specimens obtained from our patient cohort and described the composition and type of mucin present in each sample. A metagenomic analysis of the samples was performed to investigate the bacterial composition of the PMP microbiome. Secreted mucins 2 and 5AC and membrane-associated mucin-1 were the primary components of mucin in both cellular and acellular tumor specimens. The metagenomic study revealed a predominance of the phylum *Proteobacteria* and the genus *Pseudomonas*. Notably, *Pseudomonas plecoglossicida*, a species not previously reported in the human microbiome, was found to be the most abundant organism in the mucin of pseudomyxoma peritonei. Our findings suggest that the presence of MUC-2 and mucin colonization by Pseudomonas are characteristic features of both cellular and acellular disease. These results may have significant implications for the diagnosis and treatment of this rare entity.

## 1. Introduction

Pseudomyxoma peritonei (PMP) is a rare syndrome characterized by the buildup of mucin in the peritoneal cavity, often resulting from ruptured appendiceal mucinous neoplasms. While ovarian involvement is common in this condition, it is usually metastatic in nature [1]. Ovarian cystic teratoma is the only tumor of ovarian origin identified as a likely cause of PMP. Although less common, gastrointestinal mucinous adenocarcinomas and urachal cancer have also been identified as potential origins of PMP [2].

According to the Peritoneal Surface Oncology Group International (PSOGI) classification, acellular mucin is characterized by an absence of tumor epithelial cells. In contrast, PMP containing neoplastic epithelial cells in the mucin can be classified into three types based on histopathologic features and the volume of tumor cells [3]:

- Low-grade mucinous carcinoma peritonei: characterized by low-grade cytology, few mitoses, and scant mucinous tumor epithelium (<20% of tumor volume).
- High-grade mucinous carcinoma peritonei is characterized by the presence of at least one of the following features: high-grade cytology, infiltration of adjacent tissues, invasion of vascular lymphatic vessels or surrounding nerves, cribriform growth, or extensive mucinous tumor epithelium (>20% of tumor volume).
- High-grade mucinous carcinoma peritonei with signet ring cells: characterized by the presence of neoplastic signet ring cells (signet ring cells ≥ 10%).

Furthermore, the Ki-67 proliferation index has recently been proposed as a tool for stratifying high-grade PMP and predicting prognosis [4].

While the classification and prognosis of patients with PMP depends on the aforementioned histopathologic features, mucin itself has unique characteristics that warrant further study. Despite the current treatment option of cytoreductive surgery and hyperthermic intraperitoneal chemotherapy (HIPEC) [3,5], tumor recurrence and progression are frequent, with high mortality rates [5]. Therefore, identifying new therapeutic targets is crucial.

Our research aims to investigate the proteomic and biological characteristics of mucinous material in PMP as well as its metagenomic features (i.e., microbiome). We believe that a fuller understanding of mucin may provide insight into the development and progression of the disease, potentially leading to new treatment options for patients.

## 2. Materials and Methods

Patients: We obtained mucin samples from patients diagnosed with PMP who underwent surgery at Fundación Jiménez Díaz University Hospital from April 2016 to July 2020. All patients received information on the study and provided written consent to participate. The study protocol was approved by the Ethics Committee for Clinical Research of Fundación Jiménez Díaz University Hospital (PIC 75/2016_FJD). The animal study protocol was approved by the Committee on Animal Ethics and Welfare of the Fundación Jiménez Díaz University Hospital Research Institute (PIC 63/2016_FJD). We proposed a pilot study comprising a sample of nine patients who underwent surgical cytoreduction combined with HIPEC. In eight cases, the origin of the PMP was a low-grade appendiceal mucinous neoplasm (LAMN), while in one case a mucinous adenocarcinoma of the colon was the source. Following the postoperative histopathologic study, five of the PMP cases were diagnosed as acellular pseudomyxoma, and four were diagnosed as low-grade peritoneal mucinous carcinoma. In most of the patients included in our study, PMP originated from a perforated LAMN. Patients who underwent successful complete cytoreduction have remained alive without relapse. However, there was one patient who had incomplete cytoreduction. Unfortunately, this patient relapsed 6 months after the incomplete cytoreduction and eventually died within 17 months (Table 1).

Subsequently, all histopathologic analyses were repeated to detect neoplastic cells in mucin samples. Six consecutive patients were included in the final sample: three with acellular mucin and three with neoplastic cells in the mucin. The multi-step process followed to characterize mucin is described below.

**Figure 1:**
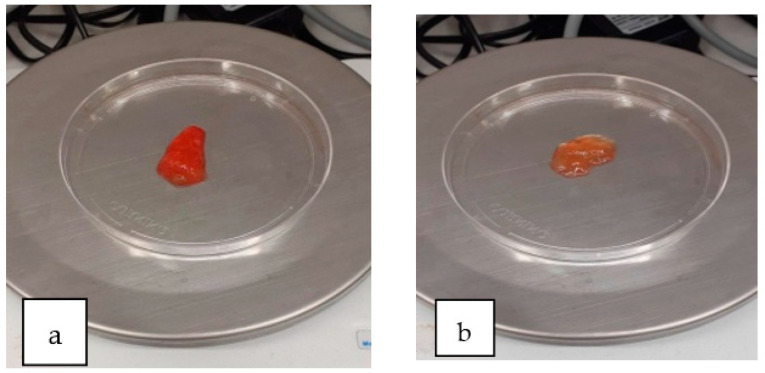
(**a**) Hard mucin; (**b**) soft mucin.

Mucin degradation: The viscosity of the mucinous component in PMP is related to such characteristics of mucin as protein concentration or cellularity (higher cellularity and protein concentration, greater sclerosis) and other external factors related to the microenvironment such as hyperosmolarity, pH < 4, or the existence of trefoil factors (soluble peptides secreted by goblet cells of the digestive tract that promote mucin viscosity) [6]. Mucolytics such as bromelain and N-acetylcysteine can be used to digest both soft and hard mucin (Figure 1) [7,8]. Soft mucin is easily degradable, while hard mucin exhibits greater sclerosis and an increased resistance to degradation [7].

Soft mucin was digested with a solution consisting of 0.3 mg/mL bromelain and 2% N-acetylcysteine and left to incubate for 90–120 min at 37 °C. Hard mucin required a longer incubation time to degrade (≤240 min).

Proteomic analysis: Following mucin digestion with bromelain and N-acetylcysteine, proteins were extracted using RIPA lysis buffer (Tris-HCl (50 nM), NaCl (150 mM), EDTA (1 mM), Nonidet P-40 (1%), DOC (0.5%), and SDS (80.1%)). The proteins were then quantified by Coomassie Brilliant blue R-250, running the gel at 100 V for 1.15 h. Finally, 20 µL per well was loaded into a precast gel (Mini-Protean TGx 4–15%, Bio-Rad, Hercules, CA, USA) using 4× Laemmli sample buffer (Bio-Rad, Hercules, CA, USA) and 5% β-mercaptoethanol as loading buffer, according to manufacturer recommendations. Subsequently, each membrane was incubated overnight with specific antibodies against the different mucins: MUC-1 (Proteintech/Fisher Scientific, Madrid, Spain), MUC-2 (ABCore Ramona-San Diego County, CA, USA), MUC-3 (Santa Cruz Biotechnology, Heidelberg, Germany), MUC-5AC (Cloud-Clone Corp/Biogen Científica, Madrid, Spain), MUC-13 (Santa Cruz Biotechnology, Heidelberg, Germany), and MUC-16 (Santa Cruz, Biotechnology, Heidelberg, Germany) at 4 °C and washed 4× with TTBS under gentle agitation for 15 min; goat anti-mouse secondary antibody (Southern Biotech/Bionova, Madrid, Spain) was added (MUC 2, 3, 13, and 16) as well as goat anti-rabbit (Southern Biotech) (MUC 1, 5AC), incubating for 1 h at room temperature under agitation. The membranes were washed with TTBS for 15 min and analyzed in an iBright system (Thermo Fisher, Madrid, Spain).

Microbiome analysis: The prokaryotic 16S ribosomal RNA (rRNA) gene is frequently used in metagenomic surveys of microbial populations due to its conserved and variable regions, which facilitate sequencing and phylogenetic classification. The microbiota in human and mouse biospecimens can be effectively studied through targeted amplification of bacterial 16S rRNA genes [9]. To identify the bacteria present in cellular and acellular mucin specimens, we performed 16S gene sequencing on the DNA extracted from these samples. Subsequently, we inoculated the mucin samples into both immunocompetent and immunocompromised mice to study the behavior of the microbiome in these experimental models.

Generation of 16S amplicons and amplicon sequencing was performed using the Illumina Miseq platform in the Genomics Unit of the Madrid Scientific Park. An initial PCR was performed with the Q5^®^ Hot Start High-Fidelity DNA Polymerase enzyme (New England Biolabs, Barcelona, Spain) using 300 pg of DNA. The primers used amplified the V3-V4 region of 16S and add extra sequences on which the second PCR was performed: 5′-ACACTGACGACATGGTTCTACA CCTACGGGNGGCWGCAG-3′ and 5′-TACGGTAGCAGAGACTTGGTCTGACTACHVGG GTATCTAAT CC-3′. Cycling of the first PCR was performed as follows: 1 × 98 °C 30 s; 23 × (98 °C 10 s, 50 °C 20 s, 72 °C 20 s); and 1 × 72 °C 2 min.

We performed a second PCR on the amplification products of the first PCR using the Q5^®^ Hot Start High-Fidelity DNA Polymerase enzyme, with the following primers (5′-AATGATACGGCGACCACCGA GATCTACACTGACGACATGGTTCTACA-3′ and 5′-CAAGCAGAAGACGGCATACGAGAT-[10 nucleotides] -TACGGTAGCAGAGACTTGGTCT-3′) from Fluidigm (Illumina Sequencers, Madrid, Spain). Cycling of this PCR was as follows: 1 × 98 °C 30 s; 14 × (98 °C 10 s, 60 °C 20 s, 72 °C 20 s); and 1 × 72 °C 2 min.

The final products were quantified by Bioanalyzer (Agilent Technologies, Santa Clara, CA, USA) to prepare an equimolecular pool that was subsequently purified by selecting the band of interest in an agarose gel with SYBR Gold (Thermo Fisher). After, the pool of amplicons was quantified by qPCR using the Kapa SYBR FAST qPCR kit for Light Cycler 480 master mix and a reference library belonging to the Genomics Unit of the Madrid Scientific Park.

Finally, the pool of amplicons was sequenced with the Illumina Miseq platform following the manufacturer’s instructions, in a paired-end (2 × 300 bp) sequencing run using MiSeq reagent kit v3-600 cycles (Illumina, Eindhoven, The Netherlands).

## 3. Results

**Figure 2:**
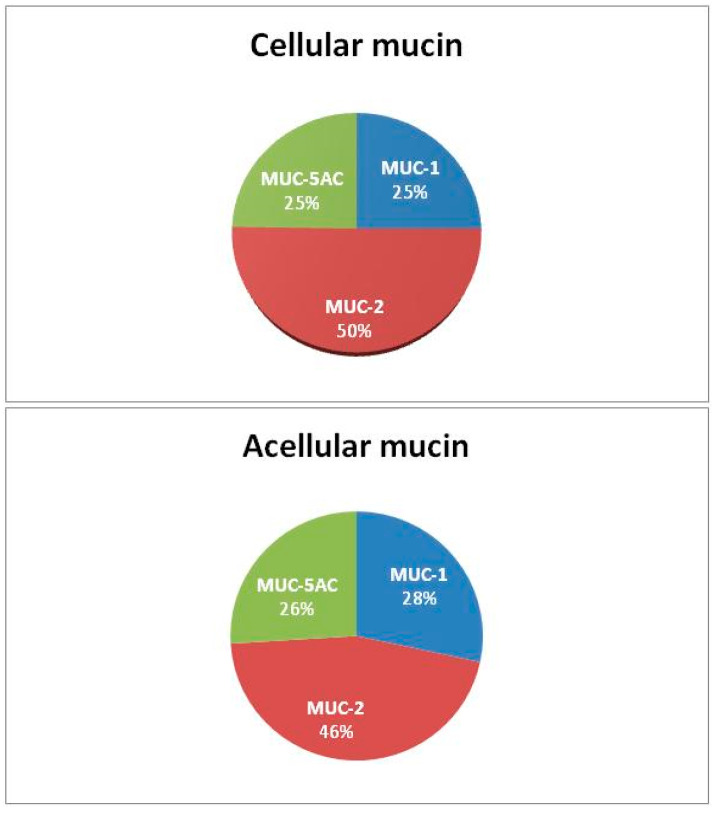
Mucin composition.

Proteomic analysis revealed the presence of secretory mucins MUC-2 and MUC-5AC, as well as the membrane mucin MUC-1, in all samples analyzed, predominantly MUC-2 (Table 2). We further detected the MUC-1 protein in mucin for the first time (MUC-1 overexpression was previously described only in tumor tissue). No other mucin types were identified, and there were no significant differences in the composition or distribution of mucin types between acellular and cellular samples (Figure 2).

**Figure 3:**
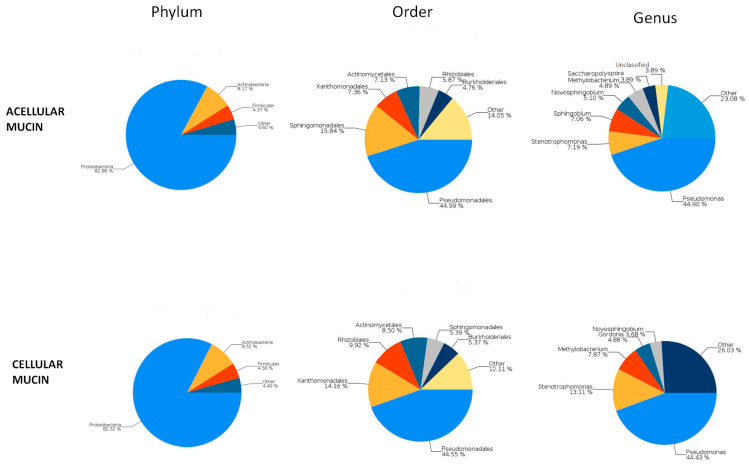
Microbiome analysis.

To examine the microbiota in mucin samples, we conducted 16S sequencing and identified different bacterial taxa. The most frequently detected phylum was genomic DNA from *Proteobacteria* in both acellular (82.86%) and cellular (82.52%) mucin, followed by *Actinobacteria* (8.17% and 8.52%, respectively). The most common bacterial order was *Pseudomonadales*, comprising 44.99% of the microbiome in acellular mucin and 44.55% in cellular mucin. The predominant genus was *Pseudomonas*, accounting for about 45% of the germs detected in both mucin groups (Figure 3).

**Figure 4:**
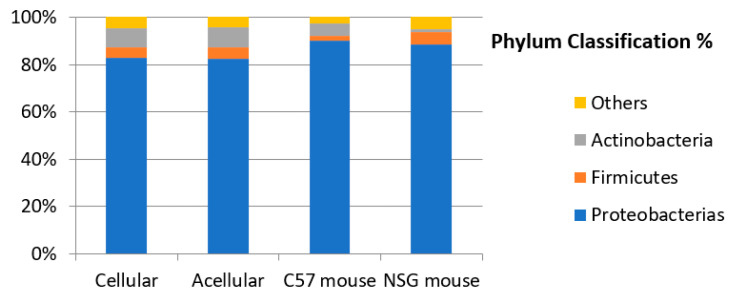

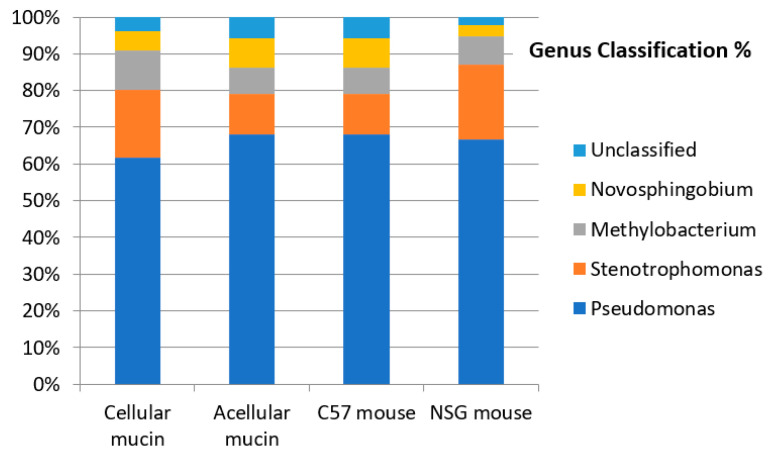
Microbiota of samples collected from patients with PMP and mucin xenoimplants in murine models.

Interestingly, the microbiota of patients with PMP and acellular mucin was almost identical to that of patients with cellular mucin. This suggests that germ colonization of accumulated mucus in PMP, primarily from *Pseudomonas*, is a specific feature of mucin independent of the patient and tumor histopathology. To confirm this hypothesis, we inoculated mucin samples into immunocompetent (C57) and immunosuppressed (NSG) mice and found that the microbiota was maintained regardless of host species and immune status (Figure 4).

*Pseudomonas plecoglossicida* was the most frequently identified bacterial species among all the samples analyzed, representing 11–21% of the total bacterial population.

## 4. Discussion

The overexpression of genes encoding different proteins of the mucin family has been described in the primary and metastatic tumor tissues of PMP. These proteins include mucin-2 (MUC-2), mucin-5AC (MUC-5AC), mucin-5B (MUC-5B), mucin-4 (MUC-4), and mucin-1 (MUC-1) [7,10,11,12,13,14,15,16,17]. The MUC-2, MUC-5AC, and MUC-5B proteins are secreted gel-forming mucins, while the MUC-1 and MUC-4 proteins are membrane-associated mucins [18]. While there is extensive research on the overexpression of mucin in tumor tissues, investigations focusing on mucin itself are considerably more limited (See Table 3 for an overview).

No reports to date have described the protein composition of acellular mucin in PMP. Our results show that the mucus in acellular mucin has a makeup that resembles the mucin of other types of PMP.

Secreted MUC-2 and MUC-5AC are the main components of mucus in PMP. MUC-5AC is expressed in the goblet cells of the gastrointestinal and respiratory epithelium as well as ovarian epithelial cells, whereas MUC-2 expression is specific to goblet cells of the intestinal epithelium [10]. MUC-2 is the only protein that has been consistently described in studies involving PMP, both those performed directly on the protein composition of mucin as well as research using tumor tissues. This pattern of mucin expression explains the appendicular origin of most cases of PMP. MUC-2 is characterized by extensive glycosylation and has been associated with mucus sclerosis and even with patient prognosis in PMP [6].

With respect to the microbiome of patients with PMP, it must be noted that the peritoneal cavity is an aseptic anatomical region. Therefore, the bacterial contamination of mucin should originate from the intestine, secondary to the perforation of appendicular mucinous neoplasms [19]. Three identifiable enterotypes have been described in the microbiome of the human gastrointestinal tract, which are defined according to the most prevalent bacterial genera: *Bacteroides* (Enterotype 1), *Prevotella* (Enterotype 2), and *Ruminococcus* (Enterotype 3) [20]. In our study, the predominant bacterial genus in the mucin was *Pseudomonas*. Therefore, we can affirm that it is not a native constituent of the digestive tract microbiome.

Gilbreath et al. [21] were the first to describe the microbiota associated with PMP and to suggest its potential impact on the pathogenesis of this disease. Different methods have been used to study the microbiota of PMP, such as cultures, in situ hybridization, and 16S sequencing. The dominant phylum described is *Proteobacteria*, with a predominance of the *Pseudomonas* genus [19], which coincides with our results.

No previous studies have specifically characterized the microbiota of acellular mucin. The results of the present analysis reveal that it has a microbiota that closely resembles that of other types of PMP. Although the presence of a microbiome having these features is not characteristic of the gastrointestinal tract, it is frequently found in the mucin within the respiratory epithelium of patients with cystic fibrosis disease. Therefore, the existence of abundant mucin and a predominance of *Pseudomonas* in the microbiome are common to PMP and cystic fibrosis. Another shared finding between these two conditions is MUC-2 overexpression, despite the fact that MUC-2 expression is specific to goblet cells of the intestinal epithelium and is not present in the respiratory epithelium under normal conditions [22]. An association between mucus hyperproduction and infection by the genus *Pseudomonas* has been described, as well as a direct relationship between the overexpression of MUC-2 and MUC-5AC and the lipopolysaccharides of this bacterial family [23,24].

Previous studies by our group demonstrated that inoculating acellular mucin in an experimental murine model could be used to reproduce acellular PMP [25]. With these findings in mind, we set out to explore the mechanism by which tumor-cell-free mucin can reproduce and grow. Based on the results of the present research, and taking into account the results of Dohrman A et al. [23] and Ben Mohamed F et al. [24], we can hypothesize that mucin overproduction may be related to colonization by bacteria belonging to the *Pseudomonas* family. To advance our understanding of PMP and improve patient outcomes, future research should investigate the relationship between the mucin microbiome and the aggressiveness and prognosis of the disease.

Findings from this and other research indicate that the microbiota may be a new therapeutic target. Preliminary results from studies that added antibiotics to the standard treatment approach for PMP (i.e., cytoreduction and HIPEC) were inconclusive, although a phase II clinical trial (NCT 02387203) is currently underway to analyze the long-term results of antibiotic administration in patients with PMP [19,26].

Limitations of the study: the primary limitations of this research are the small sample size and the exclusive use of 16S rRNA sequencing for analyzing the microbiome of mucin samples.

No previous publications have described *Pseudomonas plecoglossicida* as the most abundant *Pseudomonas* species in the mucin of PMP. This bacterium has been identified as the cause of hemorrhagic ascites in ayu fish [27], although it has never been found in the human microbiome. We can only speculate whether the occurrence of *Pseudomonas plecoglossicida* is associated with diet or some other cause. It was first identified in diseased ayus (*Plecoglossus altivelis*) [28] and has since been identified in large yellow croakers (*Larimichthys crocea*), groupers (*Epinephelus coioides*), and barramundi (*Lates calcarifer*) [29,30,31,32]. These occurrences have been documented solely in fish infections within Asian studies, and there have been no reported cases of their presence in humans. However, it is important to note that this bacterium belongs to the group of *Pseudomonas Putidas*, which are associated with human pathologies, although the transmission mechanism has not been clearly defined [33].

## 5. Conclusions

Sufficient evidence points to a direct relationship between the dominant microbiome of the pseudomyxoma and the production of mucus in PMP.

## Figures and Tables

**Table 1:** Clinical and histological features.

Age	Sex	Primary Tumor	Perforated	PMP Classification	Preoperative Chemotherapy	Cytoreduction Score	Current Status	Overall Survival (Months)
59	♂	LAMN	Yes	Metachronous LMCP (LMCP-1)	No	CC0	AWR	84
44	♀	LAMN	Yes	Synchronous AM (AM-1)	No	CC0	AWR	79
45	♂	LAMN	Yes	Metachronous AM (AM-2)	No	CC0	AWR	75
75	♀	LAMN	Yes	Metachronous AM (AM-3)	No	CC0	AWR	72
73	♀	MCA	No	Metachronous LMCP (LMCP-2)	No	CC0	AWR	54
80	♀	LAMN	No	Synchronous LMCP (LMCP-3)	No	CC1	DWR	17

Low-grade appendiceal mucinous neoplasm (LAMN). Mucinous colonic adenocarcinoma (MCA). Low-grade mucinous carcinoma peritonei (LMCP). Acellular mucin (AM). CC0: completed cytoreduction. CC1: residual tumor nodules < 0.25 cm. Alive without recurrence (AWR). Dead with recurrence (DWR).

**Table 2:** Proteomic analysis.

Patients	Samples	Mucin-1 (µg/µL)	Mucin-2 (µg/µL)	Mucin-5AC (µg/µL)
LMCP-1	Cellular mucin	46	82	40
LMCP-2	Cellular mucin	33	77	31
LMCP-3	Cellular mucin	35	70	42
AM-1	Acellular mucin	44	68	40
AM-2	Acellular mucin	44	68	40
AM-3	Acellular mucin	43	75	40

Low-grade mucinous carcinoma peritonei (LMCP). Acellular mucin (AM).

**Table 3:** Main research focused on characterizing mucin types in tumor tissue or mucin samples.

	SAMPLES	MUC-2	MUC-5AC	MUC-5B	MUC-1	MUC-6	MUC-4
O’Connell (2002) [7]	Appendix, ovarian, and peritoneal tissues (25)	✓	✓				
Mohamed (2004) [8]	Peritoneal tissue (11)	✓			✓		
Nonaka (2006) [9]	Peritoneal tissue (42)	✓	✓				
Mall (2007) [10]	Mucin (cellular)	✓	✓	✓			✓
Ferreira (2008) [11]	Ovarian tissue (28)	✓			✓		
Baratti (2009) [12]	Peritoneal tissue (85)	✓	✓				
Guo (2011) [13]	Appendix, ovarian, and peritoneal tissues (35)	✓					
Chang (2012) [14]	Appendix tissue (22)	✓	✓				
Pillai (2017) [5]	Mucin (16)	✓	✓	✓			

✓: confirmed finding.

## Data Availability

The data that support the findings of this study are available from the corresponding author, P.V.-C., upon reasonable request.
